# A vacuum lyophilization and bacterial tablet-based method for culture medium evaluation and potential use in probiotic products

**DOI:** 10.3389/fmicb.2025.1493947

**Published:** 2025-02-10

**Authors:** Wen Cui, Liang Zhao, Yuwen Shi, Wei Feng, Xiao Chen, Hui Liu

**Affiliations:** ^1^Jinan Center for Disease Control and Prevention Affiliated to Shandong University, Jinan, Shandong, China; ^2^Department of Anesthesiology, Children’s Hospital Affiliated to Shandong University and Jinan Children’s Hospital, Jinan, Shandong, China; ^3^Department of Clinical Laboratory, Yantai Yuhuangding Hospital, Yantai, China

**Keywords:** vacuum freeze-drying, culture medium, cryoprotectant, bacterial tablet, probiotic

## Abstract

**Introduction:**

The fundamental step in microbiological detection is the preparation of culture medium. The reliability and accuracy of microbiological assay heavily relies on this step. Currently in China, the most recognized standard method for such medium evaluation is ISO 11133-2014. However, this method requires highly complicated biosafety management, detailed standard strains record-keeping and substantial working time.

**Methods and results:**

Bacterial tablet is considered to be a novel strategy for bacteria culture medium evaluation. The filter membrane as a carrier ensures uniform cell dispersion, forming a singular colony that can facilitate counting. We examined the viability and storage durability of vacuum freeze-dried bacterial tablets using a filter membrane as a carrier and utilized the results to evaluate culture medium. We found that the recipe GBSS (Glycerinum, Brain Heart Infusion, Sucrose, Sodium Glutamate) showed the highest survival rate for *Escherichia coli* in vacuum freeze-drying. As a qualified medium, the productivity of target bacterium should be greater than 0.7. A comparison of freeze-dried bacterial tablet method with ISO 11133-2014 quantitative method showed the sensitivity and specificity of this novel method were 94.1% and 88.9% respectively. The results suggested that vacuum freeze-dried bacterial tablet method had high conformity when compared to ISO 11133-2014 quantitative method (*χ*^2^ = 0.25, *p* > 0.05; Kappa = 0.75).

**Discussion:**

Hence, vacuum freeze-drying method is an integral part of preservation of bacterial strains and the preparation of related biological products. In conclusion, we have developed a novel and effective disposable product for estimating efficiency of the culture medium.

## 1 Introduction

Microbial cultivation is one of the most significant methods of microbial detection and usually used as the gold standard (Brian, 2017; Tamaki et al., 2019). The quality of the medium determines the accuracy of the microbial culture results. It is estimated that the quality of the medium is affected by the fundamental components, preparation methods, suitable packing and storage conditions (Edith et al., 2023; Bonnet et al., 2020). The utilization of substandard culture media for microbial detection will significantly affect the reliability of experimental outcomes. Hence, high-quality culture media is essential for the growth and proliferation of microorganisms (Lagier et al., 2015; Magdalena et al., 2023). It aids in the formation of typical colonies or cell morphology, which provides crucial information for microbial identification and enhances the specificity and stability of microbial detection results (Jörg et al., 2017; Alejandra et al., 2018). However, the results of the yearly blind sample testing have revealed that the testing capacity of laboratories is unstable owing to the absence of medium quality evaluation. To ensure the reliability and accuracy of microbial detection results, it is imperative to assess quality of the culture medium for each batch. As a qualified medium, the productivity of target bacterium should be greater than 0.7 (Zhou, 2024; Qiu and Zeng, 2023).

Standardized methods, such as ISO 11133-2014, are prevalently employed for the culture medium evaluation. The criteria demand that culture medium be validated with standard strains. Hence, it is imperative to undertake the resuscitation and cultivation of appropriate standard strains. Nevertheless, the preservation and management of related standard strains requires special equipment such as refrigerators and rooms, specialists for management and complicated bio safety management and records, which consumes a lot of manpower, material and financial resources (ISO, 2014). At present, many laboratories can only evaluate a sampled portion of the culture medium, making it difficult to ensure the stability of microbial test results. Thus, the performance test of the culture medium remains a significant way to address a variety of important questions concerning microbiological testing. Some rules and standards advocate the use of standard strains to provide speedy and reliable results (Luo and Chen, 2021). Indeed, standard strains play an pivotal role in laboratory evaluation and method validation (Sindhulina et al., 2022; Anthony et al., 2010). It’s worth noting that ISO 11133-2014 standard requires the preparation of quantitative standard strains for medium evaluation. Nevertheless, the preparation process for quantitative standard strains is cumbersome and costly, making it difficult to conduct efficient and large-scale evaluations of culture media. Furthermore, experimental evolution is an important indicator of the reliability of experimental results (Lenski, 2017). Meanwhile, little is known concerning the commercial products for the detection performance of culture media.

*Escherichia coli* (*E. coli*) is considered the most common standard strains for culture medium evaluation (Blount, 2015). Numerous studies have confirmed that there are various protective agents for *E. coli*, such as glycerol preservation and skimmed milk preservation (Elbing and Brent, 2018a; Elbing and Brent, 2018b; Amie et al., 2021). Recent studies have shown that the resurrection rate of the *E. coli* standard strains preserved by filter paper method was 100% in 3 years at 0°C. However, this method have significant limitations. They can only ensure the existence of viable colonies and cannot achieve quantitative preservation of bacteria (Wen et al., 2022). Additionally, the aforementioned methods possess inevitable shortcomings as methods of protection in terms of accurate quantification of bacterial numbers, test cost and stability. Meanwhile, medium assessment requires quantitative bacterial numbers. Therefore, there is an urgent need for a quantitative method to preserve *E. coli* standard strains for evaluating the quality of medium, in order to solve the problems existing in the present technology.

Considerable researches have demonstrated that vacuum freeze-drying is a process in which water is frozen into a solid state through cooling and then sublimated under vacuum (Ward and Matejtschuk, 2021; Liu and Zhou, 2021). Consequently, this method is gradually extended to the preservation of bacteria, serum and other biological agents (Attasith et al., 2022). The vacuum freeze-drying preservation method is a widely used approach for preserving microorganisms due to its numerous benefits such as extended preservation time, high viability rates and minimum variability (Kawthar and Mohammed, 2022). A promising result has been noted in freeze-drying based preservation of strains. Studies have suggested that the storage stability of *sali*var*y lactobacillus* depends on the ability of protective molecules to limit damage during freeze-drying (Maria et al., 2022). It can not only reduce microbiological damage during the vacuum freeze-drying process and enhance survival rates after vacuum freeze-drying but also improve product appearance and long-term storage stability by adding an appropriate protection agent (Bircher et al., 2018). Since the protective impact of the same protective agent on different strains varies greatly, it is critical to select stable and efficient protective agents from a large pool of protective agents.

Our research provide a vacuum freeze-dried bacterial tablet to evaluate the medium, in response to the problems of the aforementioned existing technologies. We hold the opinion that a suitable carrier is a critical step in the preparation of bacterial tablets. We have found that microporous membrane can not only block the growth of bacteria to the bottom of the membrane but also do not affect the upward penetration of nutrients through pre-experiments. In addition, the microporous membrane can make the bacteria stay above the filter membrane uniformly and randomly which can form a random distribution and attain the counting function. Furthermore, we have investigated a cryoprotectant containing Brain Heart Infusion (BHI), sucrose, sodium glutamate and glycerinum to preserve *E. coli* during vacuum freeze-drying by response surface method. Here, we have demonstrated that the vacuum lyophilized bacterial tablets can effectively preserve *E. coli* under vacuum at −20°C. And a highly specific and sensitive evaluation will be achieved when it is used in the detection of culture medium. Our results showed that the freeze-drying-based method for culture media assessment has higher specificity, sensitivity and repeatability which has the potential to become an alternative for the culture media evaluation.

## 2 Materials and methods

### 2.1 Experimental material

*Escherichia coli* (ATCC25922) was the standard strain in our laboratory. Nutrient agar, Tryptose Soya Agar (TSA) medium and Trypticase Soy Broth (TSB) medium were purchased from QIAGEN (Germany). Reagents for lyophilization protection: BHI, skimmed milk and Sodium Glutamate were purchased from *gibco* (USA); Trehalose, sucrose, ascorbic acid, glutathione and glycerol were purchased from domestic companies. Microporous membrane was purchased from *Millipore* (USA). The lyophilizer was purchased from LABCONCO (Machine model: FreeZone®Triad™2.5 L) (USA).

### 2.2 Resuscitate and culture *Escherichia coli*

The frozen glycerin bacteria-preserving tubes were removed and warmed. After dipping bacterial solution with the inoculation rings, three zones were strewn on TSA medium and incubated at 37°C for 16 to 18 h. Bacteria strains were incubated at 37°C and sub-cultured at least twice. To ensure the quality of the standard strains, *Escherichia coli* were subjected to confirmation tests by physiological, biochemical detection and PCR. The research was carried out in a licensed Biosafety II Laboratory (Microbiology Laboratory, Jinan Center for Disease Control and Prevention).

### 2.3 Determination of *Escherichia coli* growth curve

The strain ATCC25922 of *Escherichia coli* was inoculated into TSB medium configured with sterile deionized water (Milli-Q Water Purification System) at 36°C for 18 h with a rotation speed of 180 r/min. Subsequently, 2 mL of the seed solution was inoculated into 200 mL TSB medium at 36°C with a rotation speed of 180 r/min. 5 mL of bacterial culture suspension was aspirated to measure the absorbance value at 600 nm every 2 h, which was used to draw the growth curve of *E. coli*.

### 2.4 Optimization of the preparation method of vacuum lyophilized tablets

Individual colonies on TSA medium were selected to prepare the bacterial suspensions of McFarland turbidity which equated to 1.5 × 10^8^ colony-forming units per millilite (cfu/ml). 10 mL of bacterial culture suspension was mixed with 90 mL of phosphate-buffered saline (PBS), followed by serial dilutions using PBS. The bacterial suspension was diluted to a final concentration of 100 cfu/5 mL. Each dilution gradient was applied in triplicate. The bacterium were uniformly distributed onto the microporous filter membrane through a series of steps, including smearing, spraying, multipoint spiking and filtering. The bacteria tablets were affixed to the TSA medium center. Following this, the sample was incubated under 37°C for 18–24 h. Subsequently, the distribution of colonies was observed.

### 2.5 Screening of a single lyophilized protection agent

BHI and skimmed milk were sterilized at 121°C for 15 min. Trehalose, sucrose, glycerol, ascorbic acid and glutathione were filtered through 0.22 μm pore size filter as a single protective agent. A volume of 0.5 mL of the bacterial culture suspension was separately added 4.5 mL of the single cryoprotectant to obtain bacterial tablets at the concentration of 100 cfu/5 mL by filtration. The bacterial tablets were subjected to a pre-cooling process at −20°C 3 h. Subsequently, it underwent a lyophilization process at −30°C under a pressure of 0.05 mBar over 24 h. The efficacy of a singular protective agent at varying concentrations was evaluated by the viable bacteria counting method. Based on the order of protective agent effects in the one-way test, four lyophilized protective agents with superior efficacy were selected for the subsequent step of the composite test. The survival rate was counted and calculated.

The survival rate is calculated as follows:

Survival rate (%) = number of live bacteria after freeze-drying (cfu)/number of live bacteria before vacuum freeze-drying (cfu) × 100.

### 2.6 Optimization of composite cryoprotectants

The Plackett-Burman (P-B) design represents a powerful statistical tool for identifying and forecasting the primary determinants of response values and optimal values among numerous variables. The single-factor test yielded the following results: BHI, sodium glutamate, sucrose and glycerol were identified as the most effective protective agents for *E. coli*. Variables A, B, C and D were set, respectively. The concentration range of BHI was set to 50 g/L to 100 g/L, the concentration range of sodium glutamate was set to 50 g/L to 100 g/L, the concentration range of sucrose was set to 10 g/L to 50 g/L and the concentration range of glycerol was 15–20%. The survival rate of bacteria following vacuum freeze-drying was designated as the response value (Y). Based on response surface method, the optimized composite cryoprotectants were selected for *E. coli*.

### 2.7 Effect of freezing temperature on bacterial survival rate after lyophilization

Pre-freezing temperature is a key parameter in the freeze-drying process. The prepared bacterial tablets were subjected to different freezing treatments: 4°C, −20°C, −40°C, −80°C for 3 h pre-freezing. After the freezing, the tablets were placed in a freeze-drying machine immediately and lowered to −30°C at a rate of 0.25°C/min. The samples were kept under vacuum freeze-drying with a vacuum of 0.05 mBar for 24 h. The total number of viable cells was determined before freezing (P0), after freezing for 3 h (P1) and during after the vacuum freeze-drying process (P2). The lyophilized tablets were directly affixed to the center of the TSA plates and incubated at 37°C for 18 h. The survival rate (%) was calculated as P1/P0 × 100%, P2/P1 × 100% and P2/P0 × 100% and recorded as N1, N2 and N3, respectively. Three independent replicates of the values were measured at each temperature.

### 2.8 Storage conditions for freeze-dried bacterial tablets

Bacterial tablets were stored under different conditions for 30d, 90d, 180d, 270d respectively:

- Frozen at 4°C; /Frozen under vacuum at 4°C.
- Frozen at −20°C; /Frozen under vacuum at −20°C.
- Frozen at −40°C; /Frozen under vacuum at −40°C.
- Frozen at −80°C; /Frozen under vacuum at −80°C.

### 2.9 Application of lyophilized bacterial tablets in nutrient medium evaluation

A new batch of nutrient agar was configured with sterile deionized water (Milli-Q Water Purification System). Sixty batches of nutrient agar were evaluated by vacuum freeze-dried bacterial tablets and compared with ISO 11133-2014 standard quantitative method.

According to ISO 11133-2014 standard quantitative method, the operation is as follows:

The growth rate was calculated using the following equation.

$P_{R}=N_{s}/N_{o}\;\left(ISO11133-2014\;7.2.1.1\right)$

P_R_ – productivity;

Ns – total number of colonies acquired on the tested medium;

N_o_ – total number of colonies obtained on the reference medium (this total number of colonies should be 100 cfu).

Reference medium selection: TSA media.

As a qualified medium, the productivity of target bacterium should be greater than 0.7. Sensitivity and specificity were estimated by comparing the lyophilisation-based bacterial tablets with the quantitative medium evaluation method in the ISO 11133-2014 standard.

### 2.10 Statistical analysis

All experiments were carried out in triplicate. Data were subject to the normal distribution and statistically described as mean ± standard deviation, and differences between groups were compared using one-way ANOVA. The chi-square test was used to compare the sensitivity and specificity of lyophilized-based bacterial tablets and standard method (*p* < 0.05 or *p* < 0.01 indicating statistical significance). The design of the surface, analysis of the data and the establishment of the regression model were obtained by Design Expert 11.1. Other data analysis and graphing were performed using GraphPad-Prism software.

## 3 Results

### 3.1 Growth curve of *Escherichia coli*

**Figure 1:**
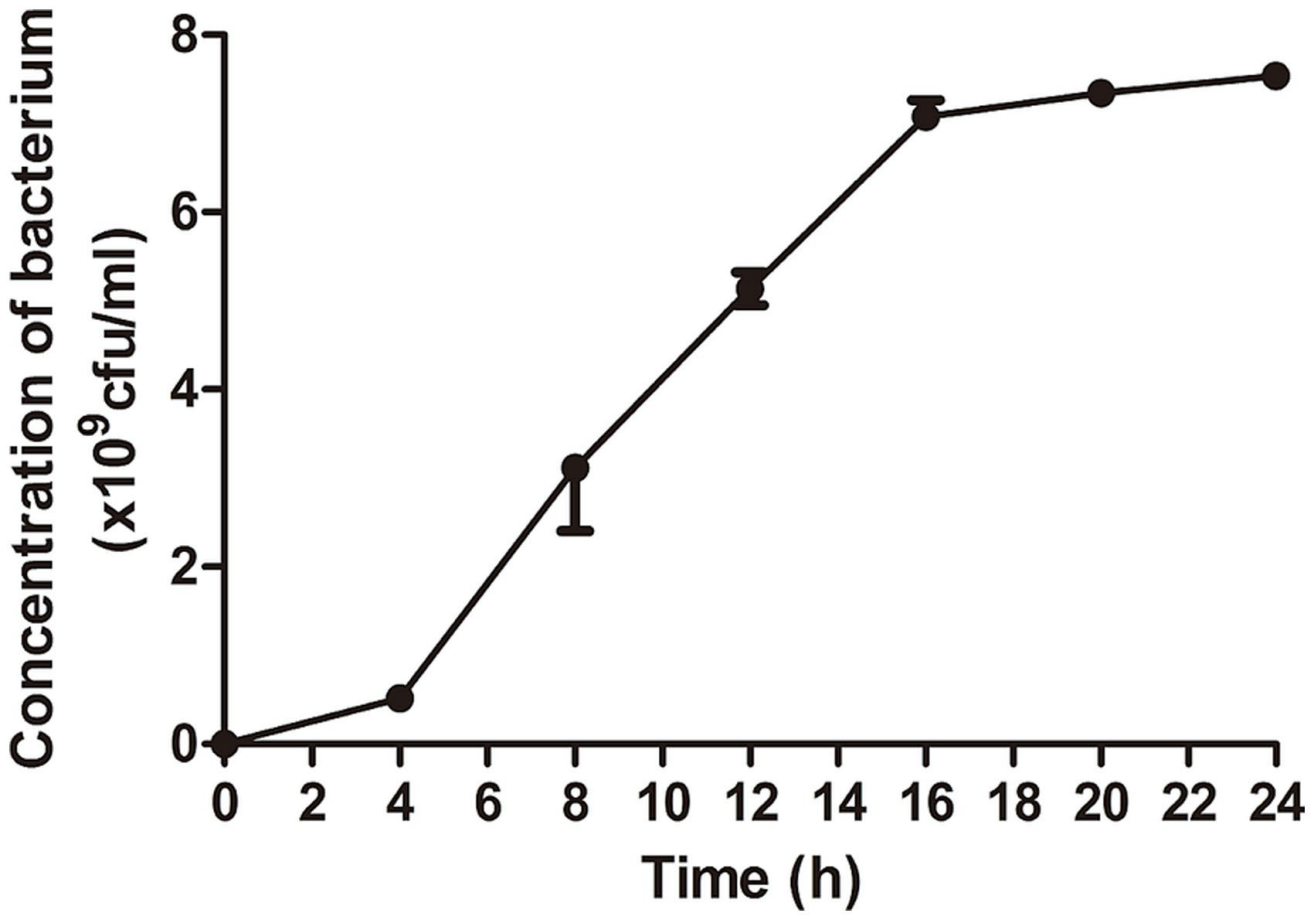
The optimal time for bacterial growth was selected for the preparation of lyophilized bacterial tablets. The dates showed that the bacterial growth entered logarithmic growth phase at 4 h and stationary phase at 16 h.

The results showed that *Escherichia coli* entered logarithmic growth phase after 4 h of culture and stationary phase after 16 h. Bacteria at the end of logarithmic phase are selected for experiments generally. Therefore, *E. coli* cultured for 16 h was chosen to prepare the lyophilized bacterial tables (Figure 1).

### 3.2 Best preparation methods of freeze-dried bacteria tablet – filtration

**Figure 2:**
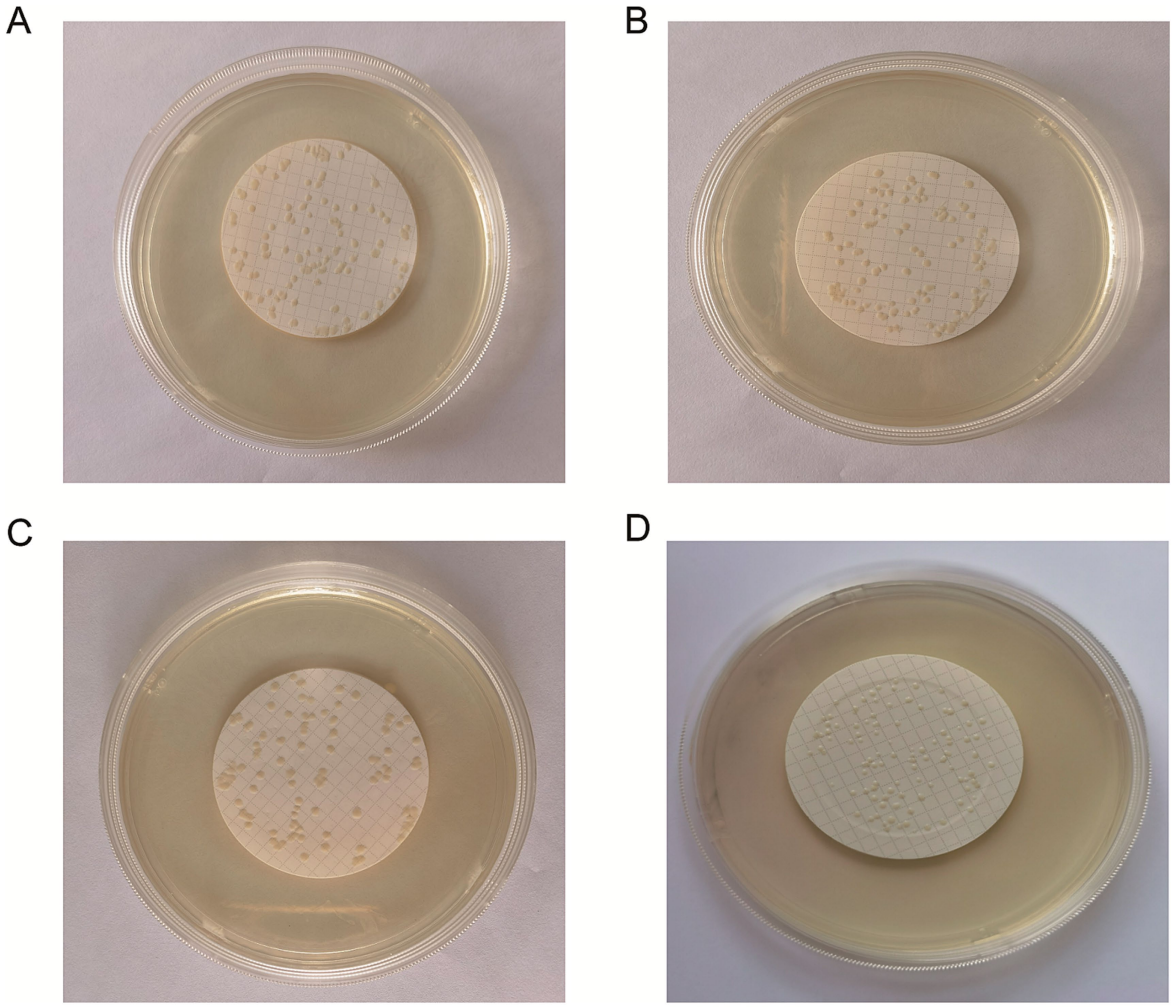
Freeze-dried bacterial tablets were prepared using the filtration method for accurate counting. The bacterial suspension was uniformly distributed onto the microporous membrane through a series of steps including smearing **(A)**, spraying **(B)**, multipoint spiking **(C)** and filtering **(D)**. The data revealed that filtration caused a greater number of uniform colonies of liquid species to pass through the filter membrane at random, improving uniformity and forming a random distribution.

The results revealed that the three methods—smearing, spraying and multi-point loading—may lead to bacterial aggregation (Figures 2A–C). A uniform dispersion of bacteria is necessary for accurate counting. Bacteria cannot exist on the edge because they would produce diffusion growth and therefore cannot be counted. To prevent bacteria from contacting the edges of the membrane, this experiment uses a specific filter. Additionally, the filtration method can improve the uniformity of the colonies and form a random distribution for the convenience of counting (Figure 2D).

### 3.3 Screening the four best single cryoprotectant (GBSS) for *Escherichia coli*

The results showed that BHI had relatively high bacterial survival rate compared to skimmed milk. At a concentration of 5% BHI, the bacterial survival rate could reach 68.1 ± 1.3%. When the concentration of sodium glutamate was 3%, the survival rate of *E. coli* could be as high as 67.4 ± 0.8%. The results of the three types of sugars at different concentrations expressed that sucrose had the highest bacterial survival rate of 65.1 ± 2.2%, followed by trehalose, with a maximum bacterial survival rate of 52.6 ± 2.0%. The lowest was glucose, with a maximum survival rate of 42.6 ± 2.0%. When glycerol concentration was 20%, the survival rate of bacteria was 64.3 ± 1.7%. The findings suggest that the bacterial survival rate in the ascorbic acid group surpass the glutathione group (Table 1).

**Table 1:** Effect of different concentrations of a single protective agent on the survival rate.

Protective agent	Concentration No.					
	1	2	3	4	5	6
BHI	64.5 ± 1.5%	60.4 ± 2.8%	63.4 ± 2.4%	65.1 ± 1.2%	68.1 ± 1.3%	63.4 ± 2.4%
Skim Milk	42.1 ± 1.2%	46.2 ± 1.5%	43.5 ± 2.8%	45.5 ± 1.6%	46.1 ± 2.6%	42.5 ± 1.4%
Sodium Glutamate	63.2 ± 2.1%	65.2 ± 1.2%	67.4 ± 0.8%	64.5 ± 0.6%	60.5 ± 2.5%	64.1 ± 0.9%
Sucrose	61.5 ± 0.7%	59.6 ± 1.1%	62.3 ± 1.5%	65.1 ± 2.2%	60.6 ± 0.7%	63.4 ± 1.3%
Trehalose	48.6 ± 1.3%	46.4 ± 0.6%	50.1 ± 2.1%	46.3 ± 1.6%	50.6 ± 1.4%	52.6 ± 2.0%
Glycerol	59.1 ± 2.4%	61.2 ± 0.5%	61.4 ± 0.6%	64.3 ± 1.7%	60.4 ± 1.5%	59.6 ± 2.1%
Ascorbic Acid	30.2 ± 0.6%	32.1 ± 2.1%	35.6 ± 0.5%	40.2 ± 1.4%	36.1 ± 2.3%	37.8 ± 1.6%
Glutathione	40.2 ± 0.8%	41.0 ± 0.4%	43.5 ± 1.6%	45.2 ± 2.4%	43.1 ± 1.8%	42.6 ± 2.4%

The concentration number (1–6) of BHI and sodium glutamate were 2, 4, 6, 8, 10, 12%; The concentration number (1–6) of skim milk were 1, 2, 3, 4, 5, 6%; The concentration number (1–6) of sucrose and trehalose were 2.5, 5, 7.5, 10, 12.5 and 15%. Glycerol concentration numbers are 5, 10, 15, 20, 25, 30%; The concentration number (1–6) of ascorbic acid and glutathione were 0.5, 1, 1.5, 2, 2.5 and 3%.

### 3.4 Optimization composite cryoprotectant for *Escherichia coli* by RSM

**Figure 3:**
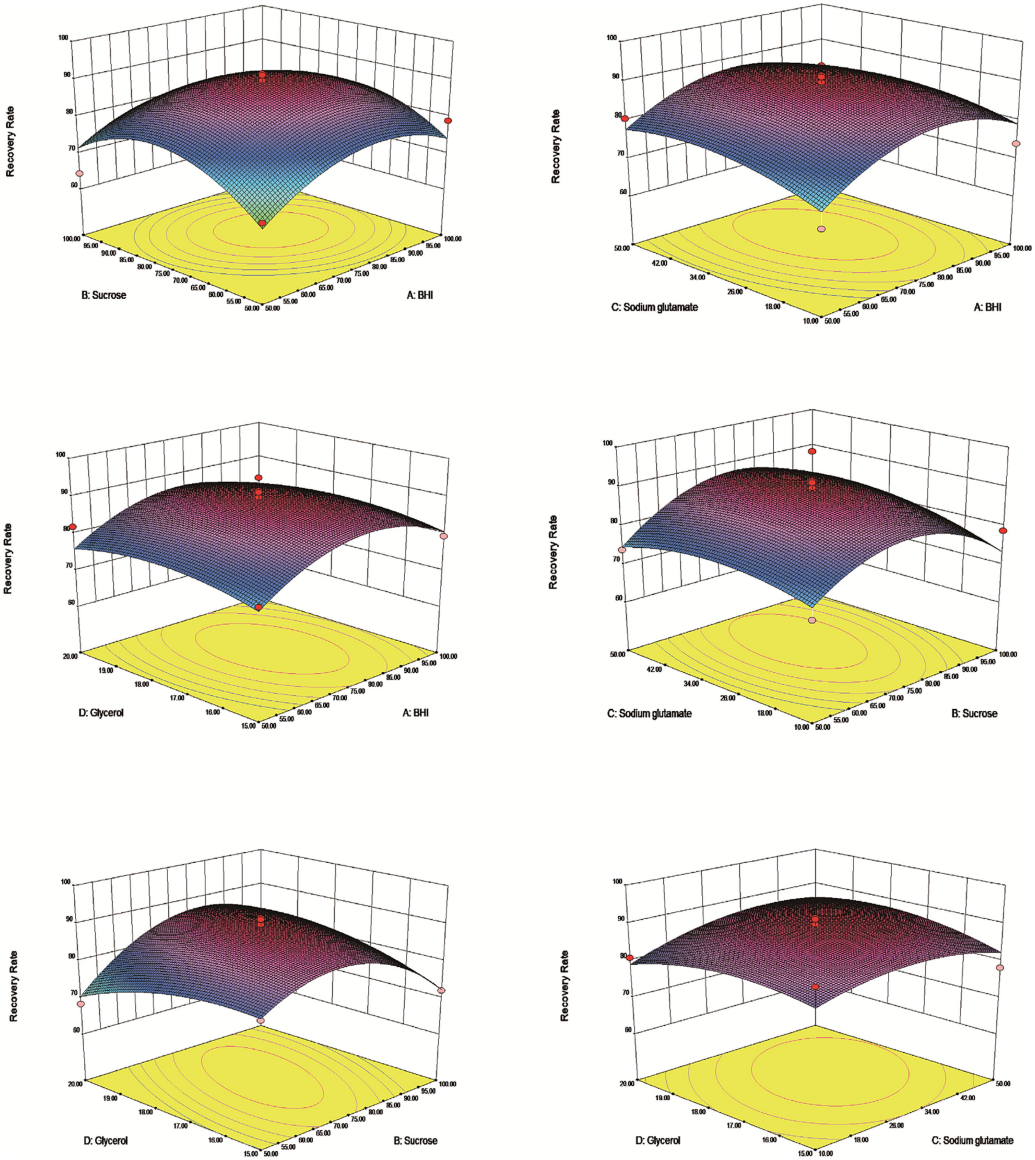
Response surface methodology of vacuum freeze-drying cryoprotectants. The dates indicated that the highest survival rate of the tablets under the protective agent was predicted to be 90.5014% at a concentration of 77.15 g/L for BHI, 77.97 g/L for sucrose, 46.19 g/L for sodium glutamate and 17.27% for glycerol.

The results demonstrated that an effective experimental model of optimized protective agents was designed through RSM to record the *E. coli* survival rate and the survival rate was up to 91.5% (Table 2). The *F* value of the response surface model was 2.81 and the *p* value was 0.00316. R^2^ level of the regression equation was highly significant, demonstrating that the experimental design was reliable. The equation Adj R^2^ was 0.475, suggesting that the regression equation had a good fitting degree (Table 3). The RSM results expressed that the images were convex upward and the highest point existed in the middle part, indicating that the experimental design had the best survival rate (Figure 3). The results above showed that the optimal protection of BHI, sodium glutamate, glycerol and sucrose could be composited. The experiment model predicted that the bacterial survival rate was 90.5014% with composite protective agent (17.27% for glycerol, 77.15 g/L for BHI, 77.97 g/L for sucrose, 46.19 g/L for sodium glutamate).

**Table 2:** Experimental design and results of response surface methodology.

Experimental parameter	BHI (g/L)	Sucrose (g/L)	Sodium Glutamate (g/L)	Glycerol (%)	Survival rate (%)
1	50	75	50	17.5	80.5
2	50	50	30	17.5	67.6
3	100	75	50	17.5	82.7
4	50	100	30	17.5	64.3
5	50	75	30	20	81.9
6	50	75	30	15	75.9
7	75	75	30	17.5	91.1
8	100	75	30	15	79.4
9	75	100	30	15	72.1
10	75	100	30	20	80.5
11	75	75	30	17.5	89.7
12	75	75	50	15	89.2
13	75	100	10	17.5	78.9
14	75	75	30	17.5	91.5
15	75	50	50	17.5	73.9
16	75	75	10	15	87.3
17	100	50	30	17.5	78.9
18	75	50	10	17.5	76.8
19	75	100	50	17.5	88.3
20	75	75	10	20	87.9
21	100	75	30	20	83.8
22	100	100	30	17.5	69.6
23	75	50	30	15	79.3
24	75	75	50	20	83.7
25	100	75	10	17.5	74.0
26	75	50	30	20	72.4
27	75	75	30	17.5	86.4
28	75	75	30	17.5	89.0
29	50	75	10	17.5	68.3

**Table 3:** ANOVA table for response surface methodology.

Source	Sum of squares	df	Mean Square	*F* value	*p* value
Model	1183.48	14	84.53	2.81	0.00316
A BHI	74.58	1	74.58	2.48	0.1379
B Sucrose	15.98	1	15.98	0.53	0.4783
C Sodium Glutamate	37.51	1	37.51	1.25	0.2832
D Glycerol	0.34	1	0.34	0.011	0.9171
AB	9.09	1	9.09	0.43	0.5915
AC	3.14	1	3.14	0.10	0.7515
AD	0.70	1	0.70	0.023	0.8813
BC	13.52	1	13.52	0.45	0.5138
BD	93.30	1	93.30	3.10	0.1002
CD	11.60	1	11.60	0.39	0.5448
A^2^	444.89	1	444.89	14.77	0.0018
B^2^	642.62	1	642.62	21.34	0.0004
C^2^	90.63	1	90.63	3.01	0.1047
D^2^	73.25	1	73.25	2.43	0.1412
R^2^					0.737
Adj-R^2^					0.475

### 3.5 Pre-freezing temperature −20°C can effectively improve the survival rate after freeze-drying

**Figure 4:**
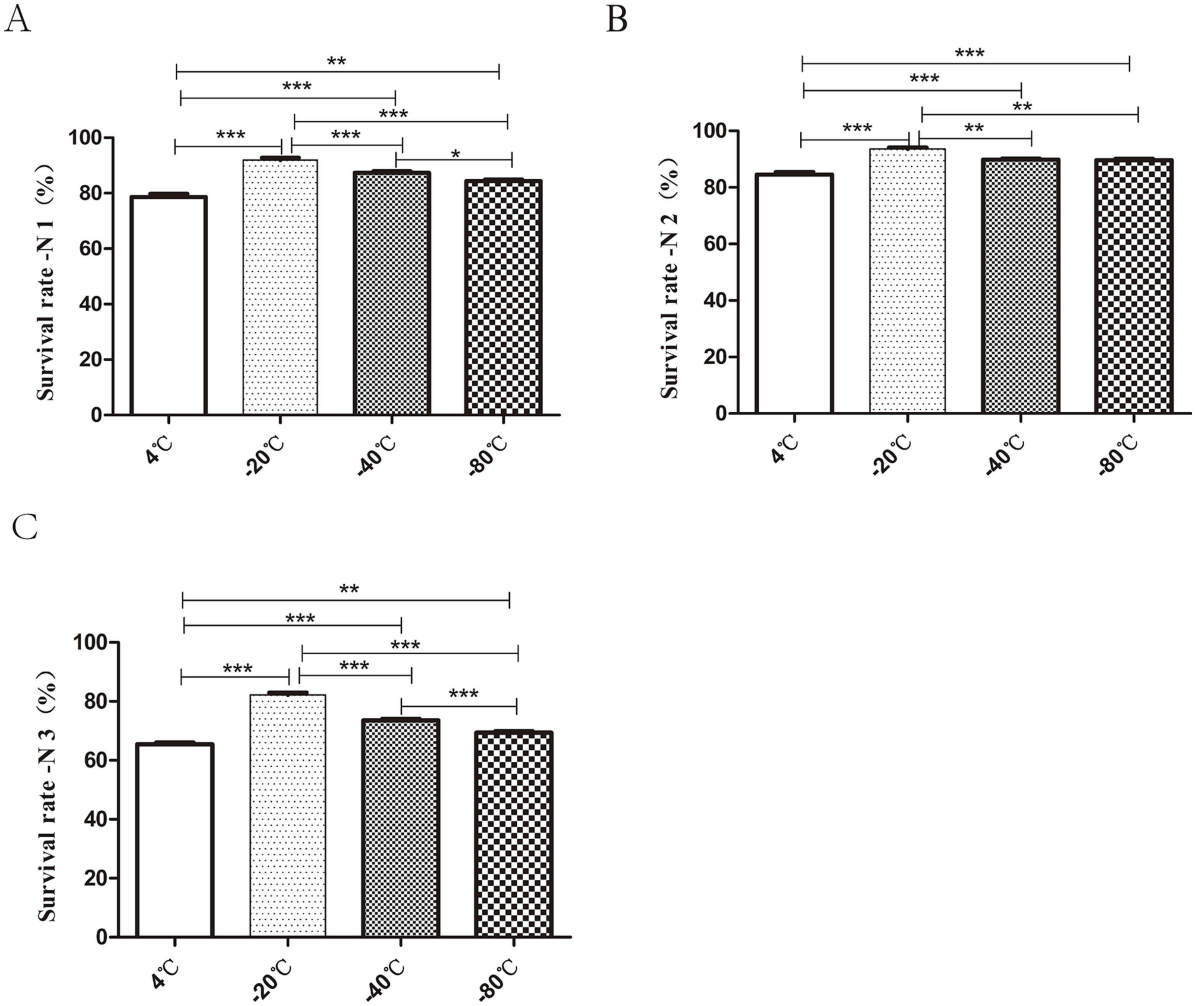
Effect of different pre-freezing temperatures on the survival of bacteria after lyophilization. The prepared tablets were subjected to different freezing treatments: 4°C, −20°C, −40°C, −80°C for 3 h pre-freezing. The total number of viable cells in each lyophilized bacterial slice was determined before freezing (P0), after freezing for 3 h (P1) and during after the freeze-drying process (P2). The survival rate (%) calculations after 3 h of freezing, and freeze-drying were expressed as P1/P0 × 100%, P2/P1 × 100%, P2/P0 × 100% and were recorded as N1 **(A)**, N2 **(B)**, N3 **(C)** respectively. In short, the highest bacterial survival rate was observed after 3 h of pre-freezing at −20°C (*p < 0.05, **p < 0.01, ***p < 0.001).

As shown in Figure 4, the strains exhibited high freezing survival rate at both −20°C and −40°C. The highest bacterial survival rate (N1) of 91.9 ± 1.17% was observed after 3 h of freezing at −20°C. The lowest bacterial survival rates (N1) were 78.6 ± 1.69% under 4°C (Figure 4A). The highest survival rate (N2) was observed at −20°C, which was about 1.1 times higher than that at −4°C (Figure 4B). The survival rate (N3) was 86.55 ± 1.01% at −20°C, less than 80% at −40°C and as low as 70% at 4°C and −80°C (Figure 4C). In conclusion, pre-freezing temperature −20°C could effectively improve the survival rate of bacteria after freeze-drying.

### 3.6 The survival rate of the bacteria is greatly improved when the storage temperature is −20°C in the vacuum state

**Figure 5:**
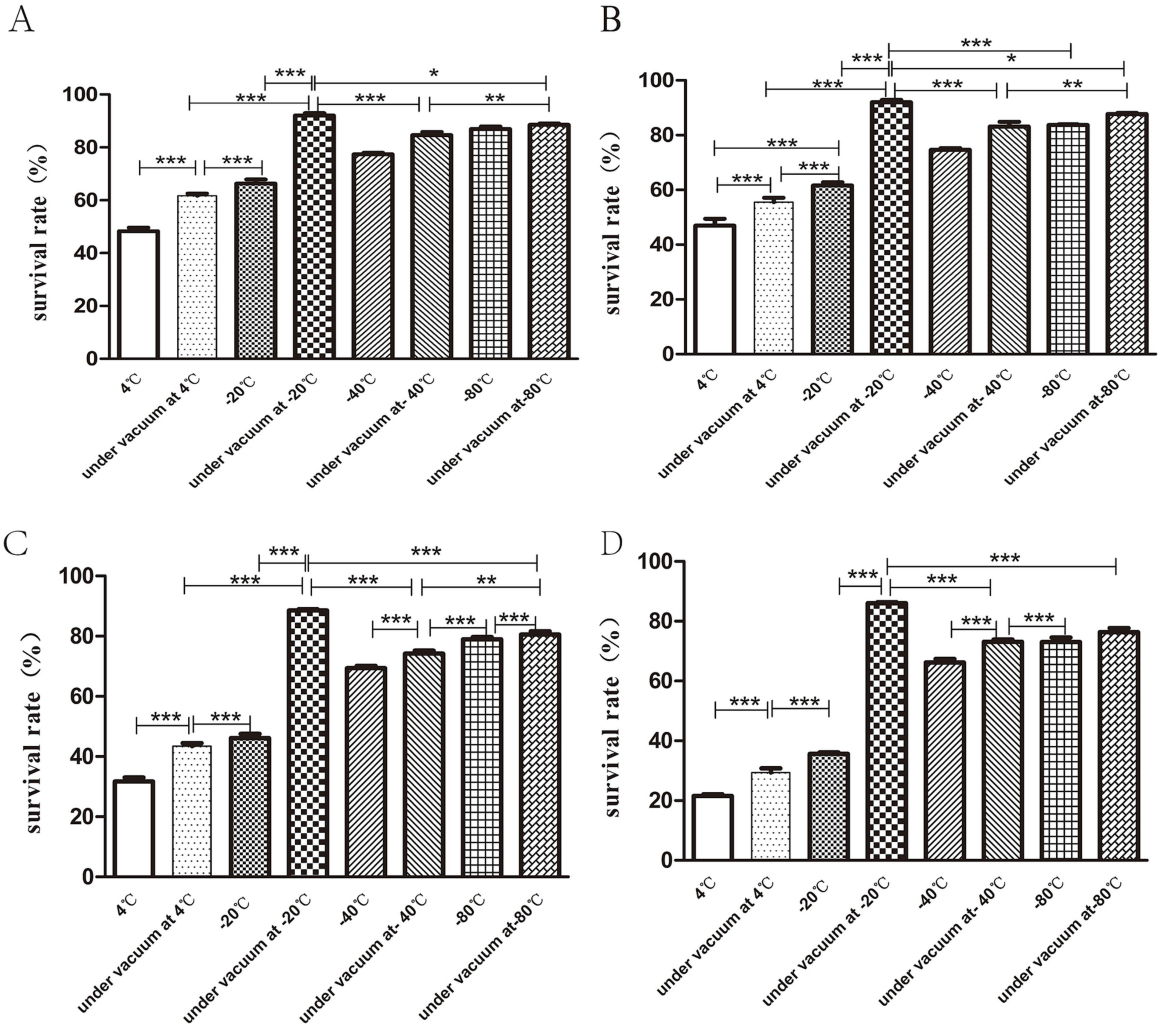
Storage conditions for freeze-dried bacterial tablets. Bacterial tablets were stored in different conditions for 30d **(A)**, 90d **(B)**, 180d **(C)**, 270d **(D)** respectively. The results showed that the survival rate of bacteria stored under vacuum at −20°Cwas the highest while the survival rate of bacteria stored at 4°C was the lowest (*p < 0.05, **p < 0.01, ***p < 0.001).

The results indicated that the highest bacterial survival rate was obtained when the freeze-dried bacterial slices were stored in vacuum at −20°C. At the same temperature, the survival rate of bacteria in vacuum preservation was higher than that in conventional preservation. When the lyophilized bacterial tablets were stored for 30 days, the survival rate of bacteria under vacuum at −20°C was 90.06 ± 1.56% and the lowest bacterial survival rate was 48.3 ± 2.25% at 4°C (Figure 5A). At 90 days of storage, the bacterial survival rate remained the highest at −20°C under vacuum, but there was no statistical significance (*p* > 0.05) between that at −80°C and −80°C under vacuum (Figure 5B). At 180 days of storage, the bacterial survival rate was markedly decreased to 31.6 ± 2.34% at 4°C (Figure 5C). At 270d of preservation, the highest survival rate of bacteria was observed at −20°C under vacuum condition, which was 86.06 ± 0.57% (Figure 5D).

### 3.7 Evaluation of culture media with vacuum lyophilized bacterial tablets

The data showed that the vacuum freeze-dried tablet method did not differ from the ISO 11133-2014 quantitative method in assessing the sensitivity and specificity of nutrient medium performance tests. 48 samples were tested as qualified by freeze-dried bacteria tablet method among the 51 samples tested as qualified by ISO 11133-2014 quantitative method and the positive coincidence rate was 94.1%; while 9 samples tested as unqualified by ISO 11133-2014 quantitative method were negative by freeze-dried bacteria tablet method and the negative coincidence rate was 77.8%. A comparison of ISO 11133-2014 quantitative method with freeze-dried bacteria tablet method showed the sensitivity, specificity, accuracy and the positive and negative predictive value were 94.1% (48/51), 88.9% (8/9), 88.9% (8/9), 97.9% (48/49) and 72.7% (8/11) (*χ*^2^ = 0.25, *p* > 0.05; Kappa = 0.75), respectively (Table 4).

**Table 4:** A comparison of ISO 11133-2014 with vacuum freeze-dried bacterial tablet method (*n* = 60) for performance test of the culture medium.

		ISO 11133-2014	
		+	−
Vacuum freeze-dried tablet	+	48	1
	−	3	8

*χ2 = 0.25, p > 0.05; Kappa = 0.75.*

## 4 Discussion

Generally, standard strains provide a reference for quality control in scientific studies. Therefore, standard strains play an important role in laboratory evaluation and method validation. A large number of literatures have reported new preservation methods for *Escherichia coli* as a standard strain. Some studies have found an inexpensive and simple method for preserving model bacteria *E. coli* in natural polymers. The data indicated that the time for *E. coli* degradated to 100 cfu/mL at 25°C was estimated to 186 days which was preserved in acacia gum (Krumnow et al., 2009). However, compared with the number of colonies before preservation, the survival rate of bacteria was relatively low. In addition, ceramic bead preservation tubes were used as containers for storing the strains. The surface of the ceramic beads has concave holes for adsorption and preservation of bacteria. The *E. coli* strains stored in porcelain bead tubes could be stored at −20°C for 1–5 years (Yang, 2020). Indeed, quantitative preservation of *E. coli* is a thorny issue. A previous study by *Bellali* showed that *E. coli* freeze-dried under a new protective agent containing 10% sucrose, 10% trehalose and 10% skim milk decreased the survival rate to 41.65% after 30 days of storage (Bellali et al., 2020). In our study, we investigated the preservation efficacy of different cryoprotectants against *E. coli* after vacuum freeze-drying.

Protective agents must be added before freeze-drying to improve the survival rate and storage stability of bacteria (Ward and Matejtschuk, 2021; Cheng et al., 2022; You et al., 2019). There is growing body of evidence that trehalose and sucrose are the most widely used cryoprotective agents (Starciuc et al., 2020). It can effectively prevent the inactivation of biologicals due to its low crystallization rate and high glass transition temperature (Zheng, 2023). In addition, trehalose has good hydration ability, which further enhances its resistance to freezing dehydration (Zhang et al., 2017). Our results suggested that the *E. coli* protection rate of trehalose was less than 60%, while sucrose was more effective. Indeed, sucrose is also an effective protective agent. This speculation is supported by the previous investigations which demonstrated that sucrose could achieve 100% survival rate against *Lactobacillus plantarum* as a single protective agent (Oluwatosin et al., 2021). A great deal of studies have found that the glass transition temperature of proteins is higher than that of sugars, suggesting that proteins play a major role in freeze drying (Dráber et al., 2021). In addition, skimmed milk can provide an additional layer of protection that can protect cell membranes (Alebouyeh et al., 2024). However, we noticed that BHI had a better protective effect for *E. coli*. Although the exact mechanisms of cell damage during the freeze-drying and storage processes for different cryoprotectants are not completely understood, the differences in *E. coli* bacterial survival rate emphasize the importance of investigating the optimal cryoprotectants for freeze-drying.

Plenty of studies have proved that different freezing parameters also induce different stress reactions to the same strains. Pre-freezing temperature also plays a key role in the vacuum freeze-drying process (Wang et al., 2019; Polo et al., 2017). It has been shown that changing the pre-freezing temperature improves the survival rate of *Lactobacillus plantarum* by altering cell membrane integrity, cell membrane permeability and lactate dehydrogenase activity (Wang et al., 2020). In the present study, we discovered that *Escherichia coli* displayed higher viability after freeze-drying when bacterial cells were frozen at −20°C than frozen at −80°C. The findings suggest that *E. coli* is more susceptible to extracellular osmotic stress. Storage temperature also affects the vitality of lyophilized cells. Studies have shown that mRNA lipid nanoparticles, after being freeze-dried, can still retain their transfection capability even after being stored at room temperature for 12 weeks (Meulewaeter et al., 2023). However, we expect that the vacuum freeze-dried bacterial tablets can be stored under standard laboratory conditions (atmospheric pressure and temperature) which is convenient for transportation and storage. In general, the most common refrigerator in the laboratory is generally four different temperatures which are 4°C, −20°C, −40°C and −80°C. Therefore, we adopted four different temperatures in order to select the best preservation conditions to achieve better preservation effect. Based on this, this study evaluated four distinct storage temperatures (4°C, −20°C, −40°C and −80°C) as well as the vacuum state at each temperature. However, the data indicated that the maximum bacterial survival rate was 90.06 ± 1.56% which was observed at −20°C under vacuum conditions.

Few methods, however, are available as routine tools for direct detection of medium evaluation although the national standard has been used for evaluation of medium. In this study, we generated a novel method for estimating efficiency of the culture medium by vacuum freeze drying. We found that protective agents including Glycerinum, BHI, Sucrose and Sodium Glutamate were the appropriate protective agents due to their high survival rate when compared with the others. Meanwhile, four protective agents (GBSS), which had better protective effect on the freeze-drying process of the bacteria, were selected for composite optimization by RSM. We observed that the composition of protective agents were 77.15 g/L for BHI, 77.97 g/L for sucrose, 46.19 g/L for sodium glutamate and 17.27% for glycerol, which had good protective effect on *Escherichia coli*. And the survival rate of *Escherichia coli* was 90.5014%. Moreover, 48 samples presented positive results among 51 qualified samples of nutrient agar medium that had been defined by ISO 11133-2014 quantitative method, with 94.1% sensitivity and 88.9% specificity. The results suggested that vacuum freeze-dried bacterial tablet method had high conformity when compared to ISO 11133-2014 quantitative method. And it indicated that vacuum lyophilized bacterial tablets had a higher test performance and might provide a novel strategy for estimating efficiency of the culture medium. We have successfully optimized composite cryoprotectants for *E. coli* which can protect the bacteria as well.

**Figure 6:**
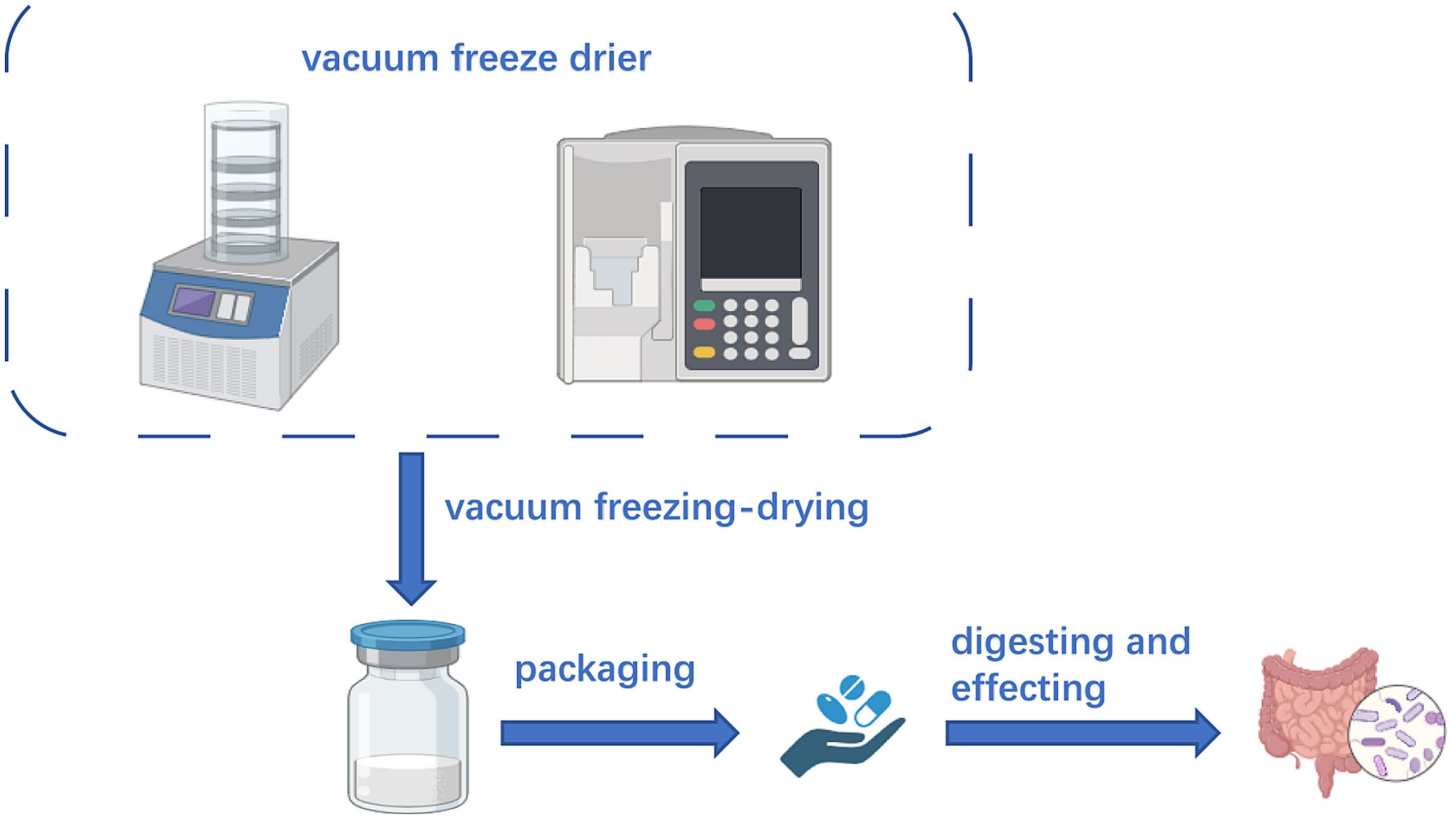
Prepared probiotics by vacuum freeze-drying method.

Cryopreservation can be seen as one of the standard methods of preserving bacteria for a long time. However, it has several disadvantages such as the need for subzero transportation and thus high energy costs. In addition, it can cause cellular damage to bacterial cells (Bircher et al., 2018). Therefore, drying techniques are preferred for preservation of bacteria. Freeze-drying provide a powerful tool that is useful for the preservation of strains (Lakshminarayana and Madhusudhan, 2018). Existing research have demonstrated that using the response surface method to optimize the cryoprotectants for freeze-dried *bifidobacterium* has been successful which can effectively protect probiotic bacteria with a survival rate of 90.37% (Chen et al., 2019). Our results have proved that the vacuum freeze-drying technology of screening cryoprotectants by response surface methodology (RSM) has obvious advantages in bacterial preservation. Furthermore, our study have explored that the survival rate of bacterial slides was 86.06 ± 0.57% after storage for 9 months. It has been reported in the literature that the drying technology of probiotics is the most critical step in developing new drugs (Broeckx et al., 2016). We have compared the drying preparation methods of probiotics by literature review, such as spray drying, vacuum drying, freeze drying, fluidized bed drying (Table 5). In these processes, multiple factors also affect the activity of probiotic-related strains (Iaconelli et al., 2015; Fu et al., 2018). In short, freeze-drying is considered the mildest dehydration method and is widely used for various probiotic powders (Fonseca et al., 2015; Kiepś and Dembczyński, 2022; Figure 6). Preparation process of probiotics is complex and challenging. Encapsulation technology is also a effective method to enhance the activity of probiotics (Šipailienė and Petraitytė, 2018; Zhao et al., 2024; Nath et al., 2024; Sharma et al., 2024). Furthermore, the protective effect of glycerol cryoprotectant cannot maintain a high survival rate of probiotic-related strains for long-term storage. It is necessary to develop a new type of cryoprotectant to better protect the probiotic-related strains during the freeze-drying process. Therefore, the use of response surface methodology (RSM) to select composite cryoprotectants for the preparation of probiotic-related strains and biological agents through vacuum freeze-drying method has potential application value.

**Table 5:** The feature of the different drying methods of probiotics.

Drying methods	Advantage	Deficiency	References
Spray Drying	Fast; Inexpensive; Uniform shape and particle size distribution	Inactivate of the bacteria at high temperatures	Sosnik and Seremeta (2015)
Vacuum Drying	Gentle temperature	Viability loss of heat-sensitive probiotics	Broeckx et al. (2016)
Fluidized Bed Drying	Short drying time	Low yields	Broeckx et al. (2016)
Vacuum Freeze-Drying	Long shelf life and stability; Protective active ingredient; Wide range of application	Longer drying time	Valentina and Estefania (2020)

Taken together, we developed a novel method for estimating efficiency of the culture medium by vacuum freeze-drying which had high sensitivity, specificity and reproducibility. The novel vacuum lyophilized bacterial tablets-based method is considered as an efficient and promising method for the evaluation of culture medium. Our study indicates that the protective agent optimized by response surface method has great potential for the preservation and transportation of lyophilized bacteria and biologics. Moreover, being the major bacteria in the evaluation of medium, *Escherichia coli* is believed to be the most common standard strain. Further work would be needed to explore the feasibility of other standard strains for the preparation of vacuum lyophilized bacterial tablets.

## Data Availability

The raw data supporting the conclusions of this article will be made available by the authors, without undue reservation.
